# Dietary antioxidant capacity and risk of type 2 diabetes mellitus, prediabetes and insulin resistance: the Rotterdam Study

**DOI:** 10.1007/s10654-019-00548-9

**Published:** 2019-08-09

**Authors:** Niels van der Schaft, Josje D. Schoufour, Jana Nano, Jessica C. Kiefte-de Jong, Taulant Muka, Eric J. G. Sijbrands, M. Arfan Ikram, Oscar H. Franco, Trudy Voortman

**Affiliations:** 1 Department of Epidemiology, Erasmus University Medical Center, PO Box 2040, 3000 CA Rotterdam, The Netherlands; 2 Department of Internal Medicine, Erasmus University Medical Center, 3000 CA Rotterdam, The Netherlands; 3 German Center for Diabetes Research, Neuherberg, Germany; 4 Helmholtz Zentrum München-German Research Center for Environmental Health, Institute of Epidemiology, Neuherberg, Germany; 5 Department of Public Health and Primary Care/LUMC Campus The Hague, Leiden University Medical Center, Leiden, The Netherlands; 6 Institute of Social and Preventive Medicine (ISPM), University of Bern, Bern, Switzerland

**Keywords:** Type 2 diabetes, Dietary antioxidant capacity, Diet, Prediabetes, Insulin resistance

## Abstract

Intake of individual antioxidants has been related to a lower risk of type 2 diabetes. However, the overall diet may contain many antioxidants with additive or synergistic effects. Therefore, we aimed to determine associations between total dietary antioxidant capacity and risk of type 2 diabetes, prediabetes and insulin resistance. We estimated the dietary antioxidant capacity for 5796 participants of the Rotterdam Study using a ferric reducing ability of plasma (FRAP) score. Of these participants, 4957 had normoglycaemia and 839 had prediabetes at baseline. We used covariate-adjusted proportional hazards models to estimate associations between FRAP and risk of type 2 diabetes, risk of type 2 diabetes among participants with prediabetes, and risk of prediabetes. We used linear regression models to determine the association between FRAP score and insulin resistance (HOMA-IR). We observed 532 cases of incident type 2 diabetes, of which 259 among participants with prediabetes, and 794 cases of incident prediabetes during up to 15 years of follow-up. A higher FRAP score was associated with a lower risk of type 2 diabetes among the total population (HR per SD FRAP 0.84, 95% CI 0.75; 0.95) and among participants with prediabetes (HR 0.85, 95% CI 0.73; 0.99), but was not associated with risk of prediabetes. Dietary FRAP was also inversely associated with HOMA-IR (β − 0.04, 95% CI − 0.06; − 0.03). Effect estimates were generally similar between sexes. The findings of this population-based study emphasize the putative beneficial effects of a diet rich in antioxidants on insulin resistance and risk of type 2 diabetes.

**Electronic supplementary material:**

The online version of this article (10.1007/s10654-019-00548-9) contains supplementary material, which is available to authorized users.

## Introduction

Oxidative stress is commonly regarded as an important contributing factor in the pathogenesis of type 2 diabetes mellitus [1]. Generally, oxidative stress is the result of an excess of reactive oxygen species (ROS), which are partially reduced forms of oxygen [2]. While ROS are considered essential for normal physiological function, an excess of ROS can lead to structural damage to important biomolecules and impairment of their function [2, 3]. A biological defense mechanism against excess ROS is formed by antioxidants. These bioactive compounds may prevent the generation of ROS or scavenge free radicals [1, 2]. Antioxidants can be endogenous, i.e. naturally occurring in the human body, such as uric acid and glutathione; or exogenous, in which case they are mainly derived from the diet [2]. Exogenous antioxidants, such as vitamin E and carotenoids, form an indispensable complementary component of the natural antioxidant defense system [4].

A high dietary intake of antioxidants may lower oxidative stress and thereby lower the risk of diseases related to oxidative stress, such as type 2 diabetes. In line with this, a higher intake of certain nutrients with antioxidative properties has been associated with a lower risk of type 2 diabetes mellitus [5, 6]. In addition, serum levels of certain antioxidants have been shown to be inversely related to plasma glucose levels and measures of insulin resistance [7, 8]. However, the majority of previous studies on this topic have investigated individual antioxidant components only, as opposed to using a comprehensive measure of total dietary antioxidant capacity. The diet can contain many components with antioxidative properties which may have additive or synergistic effects, and intake of individual antioxidants may therefore not reflect the total antioxidant capacity of the diet [9]. The concept of total dietary antioxidant capacity aims to capture overall effects of antioxidants from dietary compounds and thereby facilitates studying the effects of antioxidants in the context of complex diets [10]. Major contributors to the overall antioxidant capacity of the diet are coffee, tea, red wine and various types of fruits (blueberries, grapes, oranges) and vegetables (cabbage species, spinach, broccoli) [11, 12].

To our knowledge, only one previous study, among women only, examined the overall dietary antioxidant capacity in relation to type 2 diabetes [13]. Furthermore, dietary antioxidants have not been studied in relation to intermediate stages in the development of type 2 diabetes, such as insulin resistance or prediabetes. Therefore, we aimed to determine the association between dietary antioxidant capacity and risk of type 2 diabetes, risk of prediabetes and insulin resistance in a large population-based cohort with up to 15 years of follow-up.

## Methods

### Study design and population

The general design and objectives of the Rotterdam Study have been described in detail elsewhere [14]. In brief, the Rotterdam Study (RS) is a population-based cohort which started in 1990 with the inclusion of 7893 inhabitants of the Ommoord district in the city of Rotterdam, the Netherlands, aged 55 years or older (sub-cohort RS-I). In 2000, the cohort was extended with a second sub-cohort (sub-cohort RS-II) consisting of 3011 participants who had moved into the Ommoord district or had become 55 years of age since the inception of the first sub-cohort. A further extension of the total cohort was initiated in 2006, when 3932 residents of the Ommoord district aged 45–54 years were included in a third sub-cohort (sub-cohort RS-III). These participants were interviewed at home and received extensive physical examinations at the Rotterdam Study research facility at baseline, which are repeated every 3–4 years. The Rotterdam Study has received approval from the Medical Ethics Committee of Erasmus University Medical Center and from the review board of the Dutch Ministry of Health, Welfare and Sports. All participants have provided written informed consent [14].

### Population for analysis

Of the 14,926 participants in the Rotterdam Study, valid dietary data were available at the baseline examination round for each cohort for a total of 9701 participants [15]. Among the 5225 participants without valid dietary data, 5141 individuals had no dietary data available, and 84 were judged to have invalid dietary data because their daily energy intake did not exceed 500 kcal or was greater than 5000 kcal. Of the 9701 participants with valid dietary data, 1126 were excluded because they had prevalent cardiovascular disease (defined as a history of stroke, heart failure, myocardial infarction or revascularisation procedure) and 415 were excluded because they had prevalent cancer. Of the remaining 8160 participants, 1682 had no information on glucose status available and 682 had prevalent type 2 diabetes. Thus, our population for analysis consisted of 5796 individuals. Information on fasting serum glucose and insulin, used to calculate homeostatic model assessment of insulin resistance (HOMA-IR), was available for 5422 of these individuals.

### Dietary assessment

Dietary data were collected by means of a semi-quantitative food frequency questionnaire (FFQ), administered by a trained interviewer, during the baseline examination of the participants. For sub-cohorts RS-II and RS-I, a two-step approach was used in assessing dietary data. First, participants completed a self-administered checklist on which foods were consumed at least twice a month during the preceding year. The completed checklist was used as a basis for the structured FFQ interview, performed by a trained dietician, about consumption frequencies and amounts at the Rotterdam Study research facility. The FFQ used in these sub-cohorts consisted of 170 items and was developed for and validated among the elderly [16]. For sub-cohort RS-III-I, collection of dietary data was performed by means of a single self-administered, 389-item, semi-quantitative FFQ which was based on an existing validated FFQ developed for Dutch adults [17, 18]. Portion sizes in grams per day were estimated using standard household measures. Food intake data were subsequently converted into daily energy and nutrient intake using the Dutch Food Composition Tables of 1993 for RS-I-1, 2001 for RS-II-1, and 2006 for RS-III-1.

### Assessment of total dietary antioxidant capacity

In order to estimate the total dietary antioxidant capacity, we used the Antioxidant Food Table published by Carlsen and colleagues, who determined the antioxidant content of over 3100 types of food and beverages using a ferric reducing ability of plasma (FRAP) assay [10]. The FRAP assay measures the reduction of ferric ion (Fe^3+^) to ferrous ion (Fe^2+^) and has been used extensively in nutrition science [2, 19]. The FRAP value of each type of food extracted from the Antioxidant Food Table (mmol/100 g) was multiplied by its consumption frequency for every participant, and we then summed these values across all dietary sources of antioxidants to calculate a FRAP score for every participant representing the total dietary antioxidant capacity. Nutrition scientists from Wageningen University, the Netherlands, were consulted to determine the closest Dutch food equivalent for products that had different FRAP measurements listed for different manufacturers in the Antioxidant Food Table. No detailed data were available on the consumption of food supplements in our study, so we did not include food supplements in the calculation of the total dietary antioxidant capacity.

### Ascertainment of type 2 diabetes mellitus, prediabetes, normoglycaemia and HOMA-IR

Fasting blood samples were obtained from participants during their visit to the Rotterdam Study research facility by means of venipuncture. The samples were stored at − 80 °C in 5 mL aliquots. Glucose levels were measured using the glucose hexokinase method within 1 week of sampling [20]. In 2008, insulin levels were measured in these samples by means of electrochemiluminescence immunoassay technology using a Roche Modular Analytics E170 analyzer (Roche Diagnostics GmbH, Mannheim, Germany). We calculated HOMA-IR as the product of fasting serum glucose (mmol/L) and fasting serum insulin (mU/L) levels divided by 22.5. All measurements were performed at the clinical chemistry laboratory of Erasmus University Medical Center. We obtained data on the use of glucose-lowering medication through structured home interviews as well as pharmacy dispensing records. In accordance with WHO guidelines and the Rotterdam Study protocol, we defined type 2 diabetes as a fasting plasma glucose level ≥ 7 mmol/L, a non-fasting plasma glucose level ≥ 11.1 mmol/L or the use of blood glucose lowering medication. We defined prediabetes as a fasting plasma glucose level > 6.0 mmol/L and < 7 mmol/L, or a non-fasting plasma glucose level > 7.7 mmol/L and < 11.1 mmol/L. We defined normoglycaemia as a fasting plasma glucose level ≤ 6 mmol/L [21]. At baseline and throughout follow-up, we ascertained prediabetes and type 2 diabetes cases using records from general practitioners, hospital discharge letters and the glucose measurements performed as part of the Rotterdam Study [22]. Two physicians independently assessed all potential prediabetes and type 2 diabetes cases and consulted an endocrinologist in case of disagreement [22]. Serum glucose levels and incident cases of type 2 diabetes and prediabetes were recorded from the third examination round of the first cohort (RS-I-3) and the baseline examination rounds from the second and third cohort (RS-II-1 and RS-III-1) onwards. Hence, these rounds were used as the baseline for follow-up in our analyses.

### Covariates

We considered the following potentially confounding variables for our analyses, based on theory and previous literature: age, sex, body mass index (BMI), hypertension, dyslipidemia, highest attained level of education, degree of physical activity, smoking status, total daily energy intake, daily alcohol intake and degree of adherence to guidelines for a healthy diet. Anthropomorphic characteristics were recorded during participants’ visits to the Rotterdam Study research facility. We calculated BMI as weight in kilograms divided by squared height in meters. We defined hypertension as the use of antihypertensive medication, having a systolic blood pressure ≥ 140 mmHg or having a diastolic blood pressure ≥ 90 mmHg. Blood pressure was recorded as the mean value of two blood pressure readings at the right upper arm in sitting position, separated by 2 min, using a random-zero sphygmomanometer. We defined dyslipidemia as a serum total cholesterol level > 6.5 mmol/L or use of lipid-lowering medication. Serum total cholesterol was determined in fasting blood samples using the CHOD-PAP method (Monotest Cholesterol kit, Boehringer Mannheim Diagnostica, Germany) [23]. We determined the use of antihypertensive and lipid-lowering drugs through home interviews and consulting pharmacy dispensing records. Smoking status and the highest attained level of education were also ascertained during home interviews. We categorized participants as never smokers, former smokers or current smokers. Education level was split into four categories: primary education, lower or intermediate general education or lower vocational education, intermediate vocational education or higher general education and higher vocational education or university education. We calculated total daily energy intake (kcal/day) and daily alcohol intake (g/day) from data obtained from the FFQs. The overall dietary pattern was taken into account using a diet quality score reflecting adherence to dietary guidelines. This dietary pattern index, described by Voortman et al. [15], reflected intake of 14 food groups, including fruits and vegetables, whole grains and whole grain products, legumes, nuts, dairy, fish, tea, unsaturated fats and oils, red and processed meat, sugar-containing beverages and salt. The final index was a score ranging from 0 to 14 with a higher score reflecting a higher diet quality. The degree of physical activity was assessed by means of the LASA Physical Activity Questionnaire and a modified version of the Zutphen Study Physical Activity Questionnaire, and was expressed as metabolic equivalent of task (MET) hours per week based on time spent in light, moderate and vigorous activity [24]. To account for the use of two different questionnaires, we divided participants into quartiles of physical activity based on questionnaire-specific standard deviation scores.

### Statistical analysis

Cox proportional hazards regression was performed with total dietary antioxidant capacity as the primary independent variable and incident prediabetes or incident type 2 diabetes as the response variable. The time scale in these models is follow-up time in years to either clinical endpoint, death, loss-to-follow-up or January 1st 2012—whichever came first. As main analysis, we first investigated associations of FRAP score with incident type 2 diabetes. Subsequently, we analyzed this trajectory in more detail by investigating incident prediabetes among normoglycaemic individuals and incident type 2 diabetes among individuals with prediabetes. We used multivariable linear regression models to assess the association between FRAP score and HOMA-IR. In these linear regression models, HOMA-IR was transformed using the natural logarithm to better approximate a normal distribution. For all outcomes, we constructed models adjusted only for age, sex and cohort (model 1), models adjusted additionally for BMI, hypertension, dyslipidemia, highest level of education attained, physical activity and smoking status (model 2), and models further adjusted for degree of adherence to dietary guidelines, total daily energy intake and daily alcohol intake (model 3). We accounted for potential non-linear relations between the independent and dependent variables by including three-knot natural cubic splines in our regression models when their use resulted in a significantly better model fit. Potential effect modification by age, sex or smoking status was investigated by introducing the product of these variables and the total dietary antioxidant capacity to our regression models. We ran separate models if the interaction terms were statistically significant at the *P* < 0.10 level. As sensitivity analyses, we repeated our analyses with a modified FRAP score calculated without the contribution of coffee because some discussion remains on the bioavailability of the antioxidants found in coffee, and we also performed our analyses excluding the first year of follow-up [13]. Five-fold multiple imputation using predictive mean matching was performed to account for missing values of covariates (ranging from 0 to 4.3%). Our results are presented as pooled hazard ratios (HRs) with 95% confidence intervals (95% CIs) obtained after multiple imputation for a standard deviation increment in total dietary antioxidant capacity. Statistical analyses were performed using R version 3.4.1 (The R Foundation for Statistical Computing, Vienna, Austria).

## Results

The baseline characteristics of the total study population (n = 5796) and the subgroups of men (n = 2266) and women (n = 3530) are displayed in Table 1. The major contributors to FRAP in our study were intake of coffee, fruit, vegetables, tea and chocolate. A comparison between participants who were and were not included in the analysis of this study based on missing data is presented in Supplementary Table 1. Because we observed statistical interactions between FRAP score and sex on risk of prediabetes (*P* value for interaction 0.06) and on HOMA-IR (*P* value for interaction 0.01), we stratified all our analyses by sex. The mean (SD) FRAP score was 24.0 (9.0) for the total population, 25.1 (9.8) for men and 23.2 (8.4) for women.

**Table 1:** Baseline characteristics of the study population

	Overall (n = 5796)	Men (n = 2266)	Women (n = 3530)	*P* value for difference between sexes
Age (years)	64.2 (9.2)	63.4 (8.7)	64.6 (9.5)	< 0.001
Body mass index (kg/m^2^)	26.9 (4.1)	26.6 (3.3)	27.1 (4.5)	< 0.001
Dyslipidemia				
No	3818 (65.9%)	1640 (72.4%)	2178 (61.7%)	0.846
Yes	1978 (34.1%)	626 (27.6%)	1352 (38.3%)	
Hypertension				
No	2394 (41.3%)	940 (41.5%)	1454 (41.2%)	< 0.001
Yes	3402 (58.7%)	1326 (58.5%)	2076 (58.8%)	
Physical activity (metabolic equivalents of task—hours/week)^a^				
LASA questionnaire (RS-I and RS-II)	81.8 (57.5)	70.6 (56.2)	88.5 (57.2)	< 0.001
Zutphen questionnaire (RS-III)	45.0 (64.7)	38.7 (55.8)	52.4 (69.1)	< 0.001
Total	71.2 (63.8)	59.8 (58.9)	77.8 (62.5)	< 0.001
Education				
Primary	650 (11.2%)	183 (8.1%)	467 (13.2%)	< 0.001
Lower vocational	2398 (41.4%)	625 (27.6%)	1773 (50.2%)	
Intermediate vocational	1660 (28.6%)	827 (36.5%)	833 (23.6%)	
Higher vocational or university	1088 (18.8%)	631 (27.8%)	457 (12.9%)	
Smoking				
Never	1932 (33.3%)	397 (17.5%)	1535 (43.5%)	< 0.001
Former	2527 (43.6%)	1242 (54.8%)	1285 (36.4%)	
Current	1337 (23.1%)	627 (27.7%)	710 (20.1%)	
Dietary guideline score	6.8 (1.9)	6.3 (1.8)	7.1 (1.9)	< 0.001
Alcohol consumption (g/day)^a^	6.6 (18.1)	13.0 (23.4)	3.44 (12.3)	< 0.001
Daily energy intake (kcal/day)	2143.8 (622.4)	2436.3 (633.3)	1955.9 (537.1)	< 0.001
FRAP score	24.0 (9.0)	25.1 (9.8)	23.2 (8.4)	< 0.001

Variables are presented as mean (SD) unless otherwise indicated

^a^Variable is presented as median (interquartile range) because it did not follow a normal distribution. Differences between men and women were assessed using Student’s *T*-tests in the case of normally distributed continuous variables, *χ*^2^-tests in the case of categorical variables and Mann–Whitney *U* tests in the case of non-normally distributed continuous variables. The statistics reported above represent the dataset after multiple imputation

Of all 5796 individuals eligible for analysis, 532 developed type 2 diabetes over a mean follow-up duration of 8.1 years (incidence rate 11.4 per 1000 person-years). We observed an association between a higher FRAP score and a lower risk of type 2 diabetes, which remained statistically significant after adjusting for metabolic and socio-economic factors in model 2 (HR 0.85, 95% CI 0.76; 0.95) and further adjustment for dietary factors in model 3 (HR 0.84, 95% CI 0.75; 0.95). For incident type 2 diabetes there was no statistical interaction between dietary antioxidant capacity and sex, and indeed we observed similar effect estimates among men (HR 0.84, 95% CI 0.71; 1.00) and women (HR 0.83, 95% CI 0.70; 0.99) after adjustment for all covariates (Table 2).

**Table 2:** Associations between total dietary antioxidant capacity, risk of type 2 diabetes, risk of type 2 diabetes among prediabetics and risk of prediabetes

	Total population (n = 5796, n cases = 532)	*P* value	Men (n = 2266, n cases = 218)	*P* value	Women (n = 3530, n cases = 314)	*P* value
Incident type 2 diabetes						
Model 1^a^	0.86 (0.76; 0.96)	0.01	0.85 (0.72; 1.00)	0.05	0.87 (0.74; 1.02)	0.09
Model 2^b^	0.85 (0.76; 0.95)	0.004	0.82 (0.70; 0.97)	0.02	0.86 (0.73; 1.01)	0.07
Model 3^c^	0.84 (0.75; 0.95)	0.01	0.84 (0.71; 1.00)	0.06	0.83 (0.70; 0.99)	0.03

	Total population (n = 839, n cases = 259)	*P* value	Men (n = 398, n cases = 114)	*P* value	Women (n = 441, n cases = 145)	*P* value
Incident type 2 diabetes among participants with prediabetes						
Model 1^a^	0.84 (0.73; 0.97)	0.02	0.85 (0.70; 1.04)	0.11	0.82 (0.66; 1.04)	0.10
Model 2^b^	0.85 (0.73; 0.98)	0.03	0.83 (0.69; 1.01)	0.06	0.85 (0.68; 1.07)	0.18
Model 3^c^	0.85 (0.73; 0.99)	0.03	0.86 (0.70; 1.05)	0.13	0.81 (0.63; 1.04)	0.10

	Total population (n = 4957, n cases = 794)	*P* value	Men (n = 1868, n cases = 297)	*P* value	Women (n = 3089, n cases = 497)	*P* value
Incident prediabetes						
Model 1^a^	0.94 (0.86; 1.03)	0.17	0.85 (0.74; 0.98)	0.02	1.01 (0.90; 1.14)	0.85
Model 2^b^	0.92 (0.84; 1.01)	0.09	0.83 (0.72; 0.95)	0.01	1.00 (0.89; 1.13)	0.99
Model 3^c^	0.93 (0.84; 1.02)	0.13	0.84 (0.72; 0.98)	0.02	0.99 (0.87; 1.12)	0.87

Results are presented as hazard ratio (95% confidence interval) for a standard deviation increment in FPAP score

^a^Model 1: adjusted for age, sex and Rotterdam Study cohort

^b^Model 2: model 1 + body mass index, hypertension, dyslipidaemia, highest level of education attained, physical activity and smoking status

^c^Model 3: model 2 + degree of adherence to dietary guidelines, total daily energy intake and daily alcohol intake

Of the 839 individuals with prediabetes at baseline, 259 developed type 2 diabetes over a mean follow-up duration of 7.4 years (incidence rate 41.5 per 1000 person-years). We also found a significant association between FRAP score and incident type 2 diabetes in this subgroup (model 3; HR 0.85, 0.73; 0.99), with similar effect estimates among men and women (*P* value for interaction 0.90) (Table 2).

Over a mean follow-up duration of 7.7 years, 794 of the 4957 individuals with normoglycaemia at baseline developed prediabetes (incidence rate 20.9 per 1000 person-years). FRAP score was not significantly associated with incident prediabetes (model 3; HR 0.93, 95% CI 0.84; 1.02). However, after stratification by sex (*P* value for interaction 0.06), we observed a significant inverse association among men (model 3; HR 0.84, 95% CI 0.72; 0.98) whereas among women, we observed no association (HR 0.99, 95% CI 0.87; 1.12) (Table 2).

Finally, in the multivariable linear regression models, we observed that FRAP score was significantly inversely associated with HOMA-IR after adjustment for age, sex and cohort [model 1; regression coefficient (β) − 0.04, 95% CI − 0.06; − 0.03]. This association remained significant after adjusting for all covariates (model 3; β − 0.04, 95% CI − 0.06; − 0.03). In the analysis stratified for sex (*P* value for interaction 0.01), the association between FRAP score and HOMA-IR was significant among both men (β − 0.03, 95% CI − 0.06; − 0.01) and women (β − 0.05, 95% CI − 0.07; − 0.03), although slightly stronger among women (Table 3).

**Table 3:** Associations between total dietary antioxidant capacity and homeostatic model assessment of insulin resistance (HOMA-IR)

	Total population (n = 5422)	*P* value	Men (n = 2135)	*P* value	Women (n = 3287)	*P* value
Model 1^a^	− 0.04 (− 0.06; − 0.03)	< 0.001	− 0.03 (− 0.06; − 0.01)	0.005	− 0.06 (− 0.08; − 0.03)	< 0.001
Model 2^b^	− 0.04 (− 0.05; − 0.03)	< 0.001	− 0.03 (− 0.05; − 0.01)	0.001	− 0.05 (− 0.07; − 0.03)	< 0.001
Model 3^c^	− 0.04 (− 0.06; − 0.03)	< 0.001	− 0.03 (− 0.06; − 0.01)	0.002	− 0.05 (− 0.07; − 0.03)	< 0.001

Results are presented as regression coefficient (95% confidence interval) for a standard deviation increment in FPAP score

^a^Model 1: adjusted for age, sex and Rotterdam Study cohort

^b^Model 2: model 1 + body mass index, hypertension, dyslipidaemia, highest level of education attained, physical activity and smoking status

^c^Model 3: model 2 + degree of adherence to dietary guidelines, total daily energy intake and daily alcohol intake

In sensitivity analyses, we observed that upon exclusion of participants with less than 1 year of follow-up, the associations between dietary antioxidant capacity and incident type 2 diabetes remained significant (HR 0.86, 95% CI 0.76; 0.98) (Supplementary Table 2). However, among individuals with prediabetes, the association was no longer significant (HR 0.90, 95% CI 0.76; 1.05). Exclusion of participants with less than 1 year of follow-up did not change our conclusion with regards to incident prediabetes, which remained significantly associated with dietary antioxidant capacity only among men (HR 0.82, 95% CI 0.70; 0.97). After excluding coffee from the calculation of the FRAP score, the associations observed previously attenuated and FRAP score was no longer significantly associated with any of the outcomes (Supplementary Tables 3, 4). Finally, in stage-specific analyses of HOMA-IR, we observed similar associations of dietary antioxidant capacity with HOMA-IR among participants with normoglycaemia (β − 0.04, 95% CI − 0.05; − 0.02) and participants with prediabetes (β − 0.03, 95% CI − 0.07; 0.002) (Supplementary Table 5).

## Discussion

In this population-based cohort, we observed that a higher total dietary antioxidant capacity is associated with a lower risk of type 2 diabetes, both in the total population and among those with prevalent prediabetes. In further stage-specific analyses, we found that a higher total dietary antioxidant capacity is also associated with lower risk of incident prediabetes among men, but not among women, and with a lower HOMA-IR among both men and women.

Our results are in line with the findings of previous studies which have investigated individual antioxidant components in relation to type 2 diabetes [5, 6, 25]. Montonen and colleagues demonstrated that various types of tocopherols were associated with a reduced risk of type 2 diabetes over 23 years of follow-up [5]. Similarly, Salonen and colleagues observed that low vitamin E levels predispose individuals to developing type 2 diabetes [25]. Sluijs and colleagues found that carotenoid intake was inversely related to risk of type 2 diabetes [6]. Furthermore, our findings confirm previous studies which have found associations between dietary antioxidant capacity and measures of insulin resistance [7, 8]. Only one previous study has examined the total dietary antioxidant capacity in relation to type 2 diabetes [13]. In line with our findings, this study observed a strongly significant inverse association, but was performed among women only and did not investigate dietary antioxidant capacity in relation to stage-specific transitions from normoglycaemia to type 2 diabetes. Thus, our study is the first that investigated total dietary antioxidant capacity among both men and women in relation to incident type 2 diabetes, including intermediate endpoints such as prediabetes and insulin resistance to capture the full trajectory from normoglycaemia to type 2 diabetes.

Dietary antioxidants may directly affect glucose homeostasis in multiple ways. It has been hypothesized that oxidative stress activates the NF-κB pathway and various protein kinase pathways [26]. Activation of these pathways may inhibit signaling between insulin receptors and the glucose transport system, which contributes to the development of insulin resistance [26, 27]. Through suppressing the formation of ROS, and thereby lowering oxidative stress, dietary antioxidants may improve insulin sensitivity. Furthermore, it has been demonstrated in animal models that antioxidants can suppress apoptosis of pancreatic β-cells induced by oxidative stress [28]. Therefore, dietary antioxidants may also help in sustaining β-cell function and preventing damage to these cells.

We found that dietary antioxidant capacity was not significantly associated with risk of prediabetes in the total study population. However, we did find significant associations between dietary antioxidant capacity and incident type 2 diabetes and HOMA-IR among both participants with normoglycaemia and those with prediabetes. Because the relative contribution of pancreatic β-cell dysfunction to the pathogenesis of type 2 diabetes increases as hyperglycaemia worsens, dietary antioxidants may more strongly affect risk of type 2 diabetes among individuals with prediabetes trough preserving β-cell function rather than attenuating insulin resistance [29]. These findings also suggest that a diet with a high antioxidant capacity will exert its protective effects against type 2 diabetes regardless of whether or not prediabetes is already present. It could therefore be hypothesized that the mechanisms underlying the protective effects of dietary antioxidants are related to both early-phase phenomena in the pathogenesis of type 2 diabetes (such as insulin resistance) and later-phase phenomena (such as β-cell dysfunction). However, the exact nature of these mechanisms is currently unclear, and further research is necessary to confirm our findings.

We observed significant modification of our effect estimates by sex for some of the analyses. However, sex differences were not consistent among outcomes: the association between total dietary antioxidant capacity and incident prediabetes was significant among men, but not among women, whereas associations with insulin resistance were slightly stronger among women compared to men. The latter observation is in line with findings reported by Okubo and colleagues [8]. Potential sex differences in associations of dietary antioxidant capacity with earlier stages in the development of type 2 diabetes could be caused by differences in visceral fat mass between men and women, because visceral fat mass is positively associated with the degree of oxidative stress and differs according to sex [30, 31]. However, further research into the nature of potential sex differences is warranted, especially because we report for the first time that these appear to be stage-specific.

Our effect estimates decreased in magnitude when the contribution of coffee was excluded from the total dietary antioxidant capacity, suggesting that part of the association is explained by coffee intake. Coffee is commonly regarded as a major constituent of the total dietary antioxidant capacity. A recent study found that coffee intake captured 54% of the variation in total antioxidant intake among Norwegian women [12]. Likewise, in our study population, coffee constituted on average 49% of the total dietary antioxidant capacity. The fact that coffee forms an integral component of the total dietary antioxidant capacity probably accounts for the significant attenuation we observed in our effect estimates when coffee intake was excluded from the FRAP score. In relation to this, coffee intake has also been shown to be inversely related to risk of type 2 diabetes [32–34]. Disregarding coffee, the most important contributors to total dietary antioxidant capacity in our study were fruit and vegetables. Indeed, it has been demonstrated that increased fruit and vegetable consumption is associated with a lower risk of type 2 diabetes [35]. The findings of our study therefore further underline the putative beneficial health effects of coffee, fruit and vegetable consumption. With regards to tea and chocolate consumption, which also contributed to dietary FRAP in our study population, both of these food groups have also been associated with lower risk of type 2 diabetes [36, 37].

The main strengths of our study include its prospective design, the large sample size and the long-duration of follow-up. This enabled us to study the association between total dietary antioxidant capacity and various endpoints in the pathway from normoglycaemia to type 2 diabetes with a large pool of validated cases. We were also able to adjust for an extensive set of socio-economic, metabolic and dietary confounders, including a measure of overall diet quality, to minimize the chance of residual confounding influencing our results. However, approximately 95% of our study population was of Caucasian ethnicity, and all participants were aged 45 years and older. Therefore, caution should be taken in generalizing our results to other populations. Furthermore, we calculated the total dietary antioxidant capacity based on an antioxidant food database developed in Norway. We cannot rule out the possibility that differences between Norway and the Netherlands with regards to the geographical origin of food may have introduced error in our estimates of the true antioxidant capacity. In addition, we had no information on the cooking methods that participants used, which may also affect the antioxidant content of food. It is also conceivable that the use of different FFQs and different food composition tables in our study caused differences between participants in the assessment of their FRAP score. However, regarding the use of different FFQs, since the use of these different questionnaires coincided with the start of a new study cohort, and “cohort” was included in our analyses as a confounder, our analyses should to a large degree be adjusted for this effect. Finally, we only estimated dietary antioxidant capacity from intake of foods, and were unable to account for the use of food supplements in our study.

In conclusion, total dietary antioxidant capacity was related to a lower risk of type 2 diabetes, but not risk of prediabetes, and was inversely associated with insulin resistance in this population-based cohort of individuals aged 45 years and older. Our findings emphasize the putative beneficial health effects of a diet rich in antioxidants with regards to the prevention of type 2 diabetes. Further studies could contribute to a better understanding of the stage-specific associations we have observed and unravel underlying mechanisms.

## Electronic supplementary material

Below is the link to the electronic supplementary material.

Supplementary material 1 (DOCX 19 kb)

## Data Availability

Data can be obtained upon request. Requests should be directed towards the management team of the Rotterdam Study (secretariat.epi@erasmusmc.nl), which has a protocol for approving data requests. Because of restrictions based on privacy regulations and informed consent of the participants, data cannot be made freely available in a public repository.

## References

[1] Maritim AC, Sanders RA, Watkins JB (2003). Diabetes, oxidative stress, and antioxidants: a review. J Biochem Mol Toxicol.

[2] Benzie IFF, Choi S-W (2014). Antioxidants in Food. Adv Food Nutr Res.

[3] Linnane AW, Kios M, Vitetta L (2007). Healthy aging: regulation of the metabolome by cellular redox modulation and prooxidant signaling systems: the essential roles of superoxide anion and hydrogen peroxide. Biogerontology.

[4] Bouayed J, Bohn T (2010). Exogenous antioxidants—double-edged swords in cellular redox state. Oxid Med Cell Longev.

[5] Montonen J, Knekt P, Järvinen R, Reunanen A (2004). Dietary antioxidant intake and risk of type 2 diabetes. Diabetes Care.

[6] Sluijs I, Cadier E, Beulens JWJ, van der A DL, Spijkerman AMW, van der Schouw YT (2015). Dietary intake of carotenoids and risk of type 2 diabetes. Nutr Metab Cardiovasc Dis NMCD.

[7] Psaltopoulou T, Panagiotakos DB, Pitsavos C, Chrysochoou C, Detopoulou P, Skoumas J (2011). Dietary antioxidant capacity is inversely associated with diabetes biomarkers: the ATTICA study. Nutr Metab Cardiovasc Dis NMCD.

[8] Okubo H, Syddall HE, Phillips DIW, Sayer AA, Dennison EM, Cooper C (2014). Dietary total antioxidant capacity is related to glucose tolerance in older people: the Hertfordshire cohort study. Nutr Metab Cardiovasc Dis NMCD.

[9] Pellegrini N, Salvatore S, Valtueña S, Bedogni G, Porrini M, Pala V (2007). Development and validation of a food frequency questionnaire for the assessment of dietary total antioxidant capacity. J Nutr.

[10] Carlsen MH, Halvorsen BL, Holte K, Bøhn SK, Dragland S, Sampson L (2010). The total antioxidant content of more than 3100 foods, beverages, spices, herbs and supplements used worldwide. Nutr J.

[11] Harasym J, Oledzki R (2014). Effect of fruit and vegetable antioxidants on total antioxidant capacity of blood plasma. Nutrition.

[12] Qureshi SA, Lund AC, Veierød MB, Carlsen MH, Blomhoff R, Andersen LF (2014). Food items contributing most to variation in antioxidant intake; a cross-sectional study among Norwegian women. BMC Public Health.

[13] Mancini FR, Affret A, Dow C, Balkau B, Bonnet F, Boutron-Ruault M-C (2017). Dietary antioxidant capacity and risk of type 2 diabetes in the large prospective E3N-EPIC cohort. Diabetologia.

[14] Hofman A, Grobbee DE, de Jong PT, van den Ouweland FA (1991). Determinants of disease and disability in the elderly: the Rotterdam elderly study. Eur J Epidemiol.

[15] Voortman T, Jong JCK, Ikram MA, Stricker BH, van Rooij FJA, Lahousse L (2017). Adherence to the 2015 Dutch dietary guidelines and risk of non-communicable diseases and mortality in the Rotterdam study. Eur J Epidemiol.

[16] Klipstein-Grobusch K, den Breeijen JH, Goldbohm RA, Geleijnse JM, Hofman A, Grobbee DE (1998). Dietary assessment in the elderly: validation of a semiquantitative food frequency questionnaire. Eur J Clin Nutr.

[17] Goldbohm RA, van den Brandt PA, Brants HA, van’t Veer P, Al M, Sturmans F (1994). Validation of a dietary questionnaire used in a large-scale prospective cohort study on diet and cancer. Eur J Clin Nutr.

[18] Feunekes GI, Van Staveren WA, De Vries JH, Burema J, Hautvast JG (1993). Relative and biomarker-based validity of a food-frequency questionnaire estimating intake of fats and cholesterol. Am J Clin Nutr.

[19] Benzie IFF, Strain JJ (1996). The ferric reducing ability of plasma (FRAP) as a measure of “antioxidant power”: the FRAP assay. Anal Biochem.

[20] Neeley WE (1972). Simple automated determination of serum or plasma glucose by a hexokinase-glucose-6-phosphate dehydrogenase method. Clin Chem.

[21] World Health Organization. Definition and diagnosis of diabetes mellitus and intermediate hyperglycaemia: report of a WHO/IDF Consultation. Geneva; 2006. p. 1–50.

[22] Ligthart S, van Herpt TT, Leening MJ, Kavousi M, Hofman A, Stricker BH (2016). Lifetime risk of developing impaired glucose metabolism and eventual progression from prediabetes to type 2 diabetes: a prospective cohort study. Lancet Diabetes Endocrinol.

[23] Vitezova A, Voortman T, Zillikens MC, Jansen PW, Hofman A, Uitterlinden AG (2015). Bidirectional associations between circulating vitamin D and cholesterol levels: the Rotterdam study. Maturitas.

[24] Hofman A, Brusselle GG, Darwish Murad S, van Duijn CM, Franco OH, Goedegebure A (2015). The Rotterdam study: 2016 objectives and design update. Eur J Epidemiol.

[25] Salonen JT, Nyyssönen K, Tuomainen TP, Mäenpää PH, Korpela H, Kaplan GA (1995). Increased risk of non-insulin dependent diabetes mellitus at low plasma vitamin E concentrations: a four year follow up study in men. BMJ.

[26] Evans JL, Goldfine ID, Maddux BA, Grodsky GM (2003). Are oxidative stress-activated signaling pathways mediators of insulin resistance and beta-cell dysfunction?. Diabetes.

[27] Newsholme P, Haber EP, Hirabara SM, Rebelato ELO, Procopio J, Morgan D (2007). Diabetes associated cell stress and dysfunction: role of mitochondrial and non-mitochondrial ROS production and activity. J Physiol.

[28] Kaneto H, Kajimoto Y, Miyagawa J, Matsuoka T, Fujitani Y, Umayahara Y (1999). Beneficial effects of antioxidants in diabetes: possible protection of pancreatic beta-cells against glucose toxicity. Diabetes.

[29] Prentki M, Nolan CJ (2006). Islet β cell failure in type 2 diabetes. J Clin Investig.

[30] Blaak E (2001). Gender differences in fat metabolism. Curr Opin Clin Nutr Metab Care.

[31] Fujita K, Nishizawa H, Funahashi T, Shimomura I, Shimabukuro M (2006). Systemic oxidative stress is associated with visceral fat accumulation and the metabolic syndrome. Circ J.

[32] Mirmiran P, Carlström M, Bahadoran Z, Azizi F (2018). Long-term effects of coffee and caffeine intake on the risk of pre-diabetes and type 2 diabetes: findings from a population with low coffee consumption. Nutr Metab Cardiovasc Dis NMCD.

[33] Bhupathiraju SN, Pan A, Manson JE, Willett WC, van Dam RM, Hu FB (2014). Changes in coffee intake and subsequent risk of type 2 diabetes: three large cohorts of US men and women. Diabetologia.

[34] Gao F, Zhang Y, Ge S, Lu H, Chen R, Fang P (2018). Coffee consumption is positively related to insulin secretion in the Shanghai high-risk diabetic screen (SHiDS) study. Nutr Metab.

[35] Li M, Fan Y, Zhang X, Hou W, Tang Z (2014). Fruit and vegetable intake and risk of type 2 diabetes mellitus: meta-analysis of prospective cohort studies. BMJ Open.

[36] Jing Y, Han G, Hu Y, Bi Y, Li L, Zhu D (2009). Tea consumption and risk of type 2 diabetes: a meta-analysis of cohort studies. J Gen Intern Med.

[37] Yuan S, Li X, Jin Y, Lu J (2017). Chocolate consumption and risk of coronary heart disease, stroke, and diabetes: a meta-analysis of prospective studies. Nutrients.

