# Supplementary material for: Dietary antioxidant capacity and risk of type 2 diabetes mellitus, prediabetes and insulin resistance: the Rotterdam Study
Source: Eur J Epidemiol. 2019 Aug 9;34(9):853–61. doi: 10.1007/s10654-019-00548-9 (PMC6759671; doi:10.1007/s10654-019-00548-9)
Supplement: Supplementary file 1 — Supplementary material 1 (DOCX 19 kb) [file 10654_2019_548_MOESM1_ESM.docx]

Supplementary Table 1. Baseline characteristics of the study population, stratified by whether or not participants were included in the analysis of this study.

|  |  | Included participants  (n = 5,796) | Excluded participants  (n = 9,130) | P-value for difference |
| --- | --- | --- | --- | --- |
| Age (years) |  | 64.2 (9.2) | 66.7 (10.8) | < 0.001 |
| Body Mass Index (kg/m2) |  | 26.9 (4.1) | 27.7 (4.5) | < 0.001 |
| Dyslipidemia | No | 3,656 (63.1%) | 2,713 (29.7%) | < 0.001 |
|  | Yes | 1,922 (33.2%) | 1,832 (20.1%) |  |
| Hypertension | No | 2,328 (40.2%) | 1,535 (16.8%) | < 0.001 |
|  | Yes | 3,370 (58.1%) | 5,690 (62.3%) |  |
| Physical Activity (Metabolic Equivalents of Task- hours/week)* | LASA questionnaire (RS-I and RS-II) | 82.0 (57.4) | 67.3 (57.4) | < 0.001 |
|  | Zutphen Questionnaire (RS-III) | 42.9 (63.2) | 36.0 (61.6) | 0.018 |
|  | Total | 71.7 (63.8) | 63.5 (60.4) | < 0.001 |
| Education | Primary | 645 (11.1%) | 2,072 (22.7%) | < 0.001 |
|  | Lower vocational | 2,386 (41.2%) | 3,384 (37.1%) | 0.015 |
|  | Intermediate vocational | 1,645 (28.4%) | 2,233 (24.5%) |  |
|  | Higher vocational or university | 1,084 (18.7%) | 1,055 (11.6%) |  |
| Smoking | Never | 1,925 (33.2%) | 2,894 (31.7%) |  |
|  | Former | 2,514 (43.4%) | 3,659 (40.1%) |  |
|  | Current | 1,329 (22.9%) | 2,192 (24.0%) |  |
| Alcohol consumption (g/day)* |  | 6.6 (18.1) | 3.2 (15.6) | < 0.001 |

Variables are presented as mean (SD) unless otherwise indicated. *Variable is presented as median (interquartile range) because it did not follow a normal distribution. Differences between men and women were assessed using Student’s T-tests in the case of normally distributed continuous variables, χ^2^-tests in the case of categorical variables and Mann-Whitney U tests in the case of non-normally distributed continuous variables. Included participants are those who had valid dietary data available, did not have cancer or a history of cardiovascular disease and had information on glucose status available at baseline. The statistics presented above stem from an unimputed dataset.

Supplementary Table 2. Associations between total dietary antioxidant capacity and risk of type 2 diabetes, type 2 diabetes among participants with prediabetes and prediabetes, excluding participants with less than one year of follow-up.

| **Incident Type 2 Diabetes** | | | | | | |
| --- | --- | --- | --- | --- | --- | --- |
|  | Total population  (n = 5,738,  n cases = 505) | P-value | Men  (n = 2,236,  n cases = 203) | P-value | Women  (n = 3,502,  n cases = 302) | P-value |
| Model 1* | 0.88 (0.78 ; 1.00) | 0.04 | 0.89 (0.75 ; 1.05) | 0.17 | 0.89 (0.75 ; 1.04) | 0.15 |
| Model 2† | 0.87 (0.78 ; 0.98) | 0.03 | 0.86 (0.72 ; 1.02) | 0.08 | 0.88 (0.74 ; 1.04) | 0.13 |
| Model 3‡ | 0.86 (0.76 ; 0.98) | 0.02 | 0.87 (0.72 ; 1.04) | 0.12 | 0.85 (0.71 ; 1.01) | 0.06 |
| **Incident Type 2 Diabetes among Participants with Prediabetes** | | | | | | |
|  | Total population  (n = 821,  n cases = 244) | P-value | Men  (n = 389,  n cases = 106) | P-value | Women  (n = 432,  n cases = 138) | P-value |
| Model 1* | 0.89 (0.77 ; 1.04) | 0.15 | 0.94 (0.77 ; 1.15) | 0.54 | 0.85 (0.67 ; 1.07) | 0.17 |
| Model 2† | 0.92 (0.75 ; 1.16) | 0.53 | 0.91 (0.75 ; 1.12) | 0.38 | 0.88 (0.69 ; 1.12) | 0.29 |
| Model 3‡ | 0.90 (0.76 ; 1.05) | 0.18 | 0.93 (0.75 ; 1.15) | 0.52 | 0.83 (0.64 ; 1.08) | 0.17 |
| **Incident Prediabetes** | | | | | | |
|  | Total population  (n = 4,888,  n cases = 753) | P-value | Men  (n = 1,837,  n cases = 280) | P-value | Women  (n = 3,051 ,  n cases = 473) | P-value |
| Model 1* | 0.93 (0.85 ; 1.02) | 0.13 | 0.83 (0.72 ; 0.97) | 0.02 | 1.01 (0.89 ; 1.14) | 0.92 |
| Model 2† | 0.91 (0.83 ; 1.00) | 0.06 | 0.81 (0.70 ; 0.94) | 0.01 | 0.99 (0.88 ; 1.12) | 0.89 |
| Model 3‡ | 0.91 (0.83 ; 1.01) | 0.08 | 0.82 (0.70 ; 0.97) | 0.02 | 0.97 (0.85 ; 1.11) | 0.68 |

Results are presented as hazard ratio (95% confidence interval) for a standard deviation increment in FPAP score.*Model 1: adjusted for age, sex and Rotterdam Study cohort. †Model 2: model 1 + body mass index, hypertension, dyslipidaemia, highest level of education attained, physical activity and smoking status. ‡Model 3: model 2 + degree of adherence to dietary guidelines, total daily energy intake and daily alcohol intake.

Supplementary Table 3. Associations between total dietary antioxidant capacity and risk of type 2 diabetes, type 2 diabetes among participants with prediabetes and prediabetes, excluding the contribution of coffee.

| **Incident Type 2 Diabetes** | | | | | | |
| --- | --- | --- | --- | --- | --- | --- |
|  | Total population  (n = 5,796,  n cases = 532) | P-value | Men  (n = 2,266,  n cases = 218) | P-value | Women  (n = 3,530,  n cases = 314) | P-value |
| Model 1* | 0.89 (0.80 ; 0.99) | 0.03 | 0.88 (0.74 ; 1.04) | 0.13 | 0.90 (0.79 ; 1.03) | 0.12 |
| Model 2† | 0.97 (0.87 ; 1.07) | 0.52 | 0.82 (0.70 ; 0.97) | 0.02 | 0.99 (0.87 ; 1.13) | 0.91 |
| Model 3‡ | 0.96 (0.85 ; 1.09) | 0.54 | 0.95 (0.79 ; 1.15) | 0.61 | 0.96 (0.82 ; 1.12) | 0.57 |
| **Incident Type 2 Diabetes among Participants with Prediabetes** | | | | | | |
|  | Total population  (n = 839,  n cases = 259) | P-value | Men  (n = 398,  n cases = 114) | P-value | Women  (n = 441,  n cases = 145) | P-value |
| Model 1* | 1.00 (0.87 ; 1.14) | 0.98 | 0.99 (0.81 ; 1.22) | 0.96 | 1.01 (0.84 ; 1.20) | 0.96 |
| Model 2† | 1.04 (0.91 ; 1.18) | 0.60 | 0.99 (0.81 ; 1.21 | 0.91 | 1.06 (0.88 ; 1.28) | 0.52 |
| Model 3‡ | 1.07 (0.92 ; 1.25) | 0.38 | 1.04 (0.84 ; 1.29) | 0.71 | 1.07 (0.85 ; 1.34) | 0.57 |
| **Incident Prediabetes** | | | | | | |
|  | Total population  (n = 4,957,  n cases = 794) | P-value | Men  (n = 1,868,  n cases = 297) | P-value | Women  (n = 3,089,  n cases = 497) | P-value |
| Model 1* | 0.89 (0.82 ; 0.97) | 0.01 | 0.90 (0.78 ; 1.04) | 0.17 | 0.89 (0.80 ; 0.99) | 0.03 |
| Model 2† | 0.96 (0.88 ; 1.04) | 0.32 | 0.96 (0.83 ; 1.11) | 0.60 | 0.96 (0.86 ; 1.07) | 0.46 |
| Model 3‡ | 0.96 (0.87 ; 1.06) | 0.44 | 1.00 (0.85 ; 1.17) | 0.99 | 0.94 (0.83 ; 1.06) | 0.30 |

Results are presented as hazard ratio (95% confidence interval) for a standard deviation increment in FPAP score.*Model 1: adjusted for age, sex and Rotterdam Study cohort. †Model 2: model 1 + body mass index, hypertension, dyslipidaemia, highest level of education attained, physical activity and smoking status. ‡Model 3: model 2 + degree of adherence to dietary guidelines, total daily energy intake and daily alcohol intake.

Supplementary Table 4. Associations between total dietary antioxidant capacity and HOMA-IR, excluding the contribution of coffee.

|  | **Total population**  **(n = 5,422)** | **P-value** | **Men**  **(n = 2,135)** | **P-value** | **Women**  **(n = 3,287)** | **P-value** |
| --- | --- | --- | --- | --- | --- | --- |
| **Model 1*** | -0.04  (-0.06 ; -0.03) | < 0.001 | -0.05  (-0.85 ; -0.023) | < 0.001 | -0.03  (-0.05 ; -0.01) | 0.001 |
| **Model 2†** | -0.02  (-0.03 ; -0.002) | 0.03 | -0.03  (-0.05 ; -0.01) | 0.004 | -0.01  (-0.02 ; 0.01) | 0.57 |
| **Model 3‡** | -0.01  (-0.03 ; 0.01) | 0.21 | -0.03  (-0.06 ; -0.01) | 0.02 | 0.003  (-0.02 ; 0.02) | 0.74 |

Results are presented as regression coefficient (95% confidence interval) for a standard deviation increment in FPAP score.*Model 1: adjusted for age, sex and Rotterdam Study cohort. †Model 2: model 1 + body mass index, hypertension, dyslipidaemia, highest level of education attained, physical activity and smoking status. ‡Model 3: model 2 + degree of adherence to dietary guidelines, total daily energy intake and daily alcohol intake.

Supplementary Table 5. Associations between total dietary antioxidant capacity and HOMA-IR among participants with normoglycaemia and prediabetes.

| **Participants with normoglycaemia** | | | | | | |
| --- | --- | --- | --- | --- | --- | --- |
|  | Total population  (n = 4,614) | P-value | Men  (n = 1,752) | P-value | Women  (n = 2,862) | P-value |
| Model 1* | -0.04  (-0.05 ; -0.02) | < 0.001 | -0.03  (-0.05 ; -0.004) | 0.02 | -0.05  (-0.07 ; -0.03) | < 0.001 |
| Model 2† | -0.04  (-0.05 ; -0.02) | < 0.001 | -0.03  (-0.05 ; -0.01) | 0.01 | -0.05  (-0.06 ; -0.03) | < 0.001 |
| Model 3‡ | -0.04  (-0.05 ; -0.02) | < 0.001 | -0.03  (-0.05 ; -0.01) | 0.01 | -0.04  (-0.06 ; -0.02) | < 0.001 |
| **Participants with prediabetes** | | | | | | |
|  | Total population  (n = 808) | P-value | Men  (n = 383) | P-value | Women  (n = 425) | P-value |
| Model 1* | -0.03  (-0.07 ; 0.004) | 0.08 | -0.03  (-0.07 ; 0.02) | 0.30 | -0.06  (-0.12 ; -0.001) | 0.05 |
| Model 2† | -0.03  (-0.06 ; 0.01) | 0.10 | -0.03  (-0.07 ; 0.01) | 0.15 | -0.04  (-0.01 ; 0.01) | 0.13 |
| Model 3‡ | -0.03  (-0.07 ; 0.002) | 0.06 | -0.03  (-0.08 ; 0.01) | 0.18 | -0.05  (-0.11 ; 0.004) | 0.07 |

Results are presented as regression coefficient (95% confidence interval) for a standard deviation increment in FPAP score.*Model 1: adjusted for age, sex and Rotterdam Study cohort. †Model 2: model 1 + body mass index, hypertension, dyslipidaemia, highest level of education attained, physical activity and smoking status. ‡Model 3: model 2 + degree of adherence to dietary guidelines, total daily energy intake and daily alcohol intake.
